# Weighing Evidence from Mendelian Randomization—Early-Life Obesity as a Causal Factor in Multiple Sclerosis?

**DOI:** 10.1371/journal.pmed.1002054

**Published:** 2016-06-28

**Authors:** Alberto Ascherio, Kassandra L. Munger

**Affiliations:** Harvard T. H. Chan School of Public Health, Harvard University, Boston, Massachusetts, United States of America

## Abstract

In this Perspective, Alberto Ascherio and Kassandra Munger discuss the implications of Richards and colleagues' study exploring the role of early-life obesity in risk of multiple sclerosis.

Could prevention or correction of obesity in early life contribute to reduction of multiple sclerosis (MS) risk? Several observational studies have found that individuals who are obese in early life have about a 2-fold increased risk of MS [1–5], including two prospective studies: in a cohort of United States women, those with a body mass index (BMI) ≥ 30 kg/m^2^ at age 18 had a greater than twofold risk of developing MS than lean women [1]; and in Denmark, individuals with childhood BMI ≥ 95th percentile were 70% more likely to develop MS than those with childhood BMI < 85th percentile [2]. While such prospective study designs eliminate or minimize bias from several sources, confounding by unknown factors cannot be excluded as an explanation for the findings.

In this issue of *PLOS Medicine*, J. Brent Richards and colleagues use a mendelian randomization (MR) approach to address the question of causality between obesity and MS risk [6]. MR has been touted as a study design that further minimizes bias, as genetic variations are expected to be randomly inherited and thus not subject to confounding. Conceptually, the MR approach is straightforward—consider a variation in a hypothetical gene G that directly affects a characteristic A (e.g., body mass index), such that carriers of the variant G’ allele have higher levels of A than noncarriers. If A causes disease B (e.g., MS), but not otherwise, we would expect carriers of G’ to have a higher risk of disease B.

Richards and colleagues used summary statistics from two separate genome-wide association studies (GWAS), one comprising over 340,000 individuals (with no information on MS) to identify genetic predictors of BMI (GIANT [7]), and one comprising over 14,000 MS cases and 24,000 controls (IMSGC [8,9]; with no information on BMI) to identify whether the genetic predictors of BMI were associated with MS risk. They identified 70 single nucleotide polymorphisms (SNPs) that were predictors of BMI and available in the IMSGC. MR analysis found that one standard deviation increase in genetically determined BMI was associated with a statistically significant 41% increased risk of MS (OR = 1.41, 95% CI: 1.20–1.66, *p* = 2.72 x 10^−5^). The authors interpreted this result as providing evidence that obesity is a causal factor in MS. There are, however, a few important limitations of this study to consider.

One underlying critical assumption of MR studies is that of no pleiotropic effects. Specifically, in this MR study, that would mean that the 70 SNPs can affect the risk of MS only through their effects on BMI, i.e., none of the genes has pleiotropic effects. However, the absence of pleiotropic effects can only be assessed indirectly [10]; this assessment was done by examining whether the 70 SNPs relate to MS risk in a manner consistent with their effects on BMI (i.e., whether the effect of each of the 70 SNPs on MS risk is proportional to its effect on BMI, a method known as MR-Egger regression), and by using a weighted median estimator [10]. While these are useful approaches, neither is unbiased when there are pleiotropic effects that are correlated with the effect on BMI, which could happen, for example, if the identified SNPs affect preference for an obesogenic diet, and that this diet, rather than obesity, is causally related to MS.

Second, the genetic determinants of BMI in GIANT were derived from adult populations, including many individuals who were past the age of MS incidence, whereas we know from epidemiological studies that only obesity in early life appears to be etiologically relevant for MS. This discrepancy is important, because genetic effects on BMI are likely to be age-dependent, as recently demonstrated [11–13]. This is not surprising, because the prevalence of obesity and median BMI increase dramatically with age [12], and the effect of a genetic variant on BMI can be present early in life or may become manifest decades later, either as a consequence of aging itself or because of interactions with environmental exposures [11]. Thus, the estimate of the magnitude of the effect of genetically increased BMI on MS risk found by Richards and colleagues is likely to be biased; the direction of the bias is difficult to predict, because there is insufficient information on the age-dependency of the 70 SNPs that contribute to the effect estimate. Further, it is a general limitation of MR studies that little information can be obtained on dose–response. The genetic effect usually explains only a modest proportion of the overall variance of the characteristic of interest (e.g., in GIANT, 97 loci explain only 2.7% of the total BMI variance [7]) and predict small variations in disease risk. Meaningful relative risks have to be estimated by extrapolating the data and assuming a linear dose–response relation, which may not be appropriate.

MR studies are increasing in popularity in part because of the increasing public availability of results from GWAS such as those used by Richards and colleagues, and are sometimes presented by themselves as proof of causality for associations reported in observational studies. We agree that MR studies are an important complement to observational investigations based on directly observed exposure–disease associations, but caution should be used in interpreting the results. Many underlying assumptions cannot be tested and are rarely fully satisfied, particularly in the case of weak instruments (i.e., an instrument that is a poor predictor of the exposure of interest) and complex exposure variables such as BMI that are determined by complex gene–environment interactions [14]. Further, important public health questions such as dose–response and relevant age at exposure cannot be directly addressed. A judgment of causality should therefore be based on the totality of evidence, including the traditional criteria of Bradford Hill [15] and the results of well-conducted MR studies.

In the case of obesity and MS risk, the results of Richards and colleagues’ MR study, when interpreted in the context of temporality, strength, consistency, and plausibility demonstrated in previous observational investigations, suggest that obesity in early life is indeed causally related to MS risk and provide a further rationale for obesity prevention.

## Abbreviations

BMI: body mass index
GWAS: genome wide association studies
MR: mendelian randomization
MS: multiple sclerosis
SNP: single nucleotide polymorphism
